# Differential plastic responses to temperature and nitrogen deposition in the subalpine plant species, *Primula farinosa* subsp*. modesta*

**DOI:** 10.1093/aobpla/plab061

**Published:** 2021-09-19

**Authors:** Hyungsoon Jeong, Yong-Chan Cho, Eunsuk Kim

**Affiliations:** 1 School of Earth Sciences and Environmental Engineering, Gwangju Institute of Science and Technology, Gwangju 61005, Korea; 2 Conservation Center for Gwangneung Forest, Korea National Arboretum, Pocheon 11186, Korea

**Keywords:** Nitrogen deposition, phenotypic plasticity, population differentiation, *Primula farinosa* subsp. *modesta*, temperature

## Abstract

Future environmental changes are projected to threaten plant populations near mountaintops, but plastic responses of plant traits that are related to demographic parameters may reduce the detrimental effects of altered environments. Despite its ecological significance, little is known about the intraspecific variation of plasticity in alpine plant species such as *Primula farinosa* subsp*. modesta*. In this study, we investigated the plastic responses of plants at the early developmental stage from four *P. farinosa* natural populations in response to temperature and nitrogen deposition under laboratory conditions. Measured traits included plant survival, leaf number, rosette diameter, carbon assimilation rate and leaf chlorophyll content. In addition, we conducted a demographic survey of the natural populations to assess the plant’s performance at the early developmental stage in the field and evaluate the ecological implications of our experimental treatments. The seedling stage contributed to the projected population growth rate in natural conditions, and the growth and survival of seedlings in the field were comparable to those grown in the control treatment. In response to high temperature, plants exhibited lower survival but produced larger rosettes with more leaves. Nitrogen deposition had little effect on plant survival and plant size; however, it increased plant survival in one population and altered the effect of temperature on the carbon assimilation rate. Populations exhibited differential plasticity indexes of measured traits in response to environmental treatments. These results suggest that even though the plants suffer from high early mortality under increasing temperature, stimulated growth at a high temperature potentially contributes to the persistence of *P. farinosa* natural populations. Natural populations might face differential extinction risks due to distinctive plastic responses to altered environments.

## Teaser

Anthropogenic environmental changes are projected to threaten plant populations near mountaintops. However, it is still inconclusive whether an alpine species in different sites would exhibit a similar pattern of demographic change. We grew plants from four natural populations of *Primula farinosa* subsp. *modesta*, and compared their responses to increasing temperature and nitrogen deposition. High-temperature treatment reduced plant survival, but it stimulated plant growth. In addition, the effect of environmental treatments on plant growth differed among natural populations. Since both survival and growth contribute to population persistence, our results suggest that *P. farinosa* populations may face differential extinction risks in the future.

## Introduction

Alpine ecosystems are projected to be endangered by future climate change because they are at the edge of climate gradients and their geographical range is decreasing rapidly (Diaz and Eischeid 2007). Similarly, plant populations near mountaintops are likely to be at high risk due to altered climatic conditions. As the mountaintop area is at the edge of climate gradients, plant populations would have limited opportunity to track such environmental changes. Although the patterns of genetic differentiation and phenotypic plasticity in plant populations can ameliorate extinction risk due to changing climate (Holt 1990; Nicotra *et al*. 2010; Shaw and Etterson 2012), limited information is available on perennial herbaceous plants in alpine environments (Valladares *et al*. 2014; Matesanz and Ramírez-Valiente 2019).

Changing climate is a well-known phenomenon of anthropogenic environmental change (Parry *et al*. 2007). However, other environmental factors are also changing. For instance, nitrogen deposition due to increasing industrial activities (Galloway *et al*. 2008) results in soil eutrophication and acidification. Plants in nutrient-poor environments such as alpine or subalpine areas are expected to be highly susceptible to nitrogen deposition (Bowman *et al*. 2006). In addition, climatic conditions and nitrogen deposition might interactively influence plant communities (Porter *et al*. 2013); therefore, the evaluation of plant response to these two factors simultaneously is required.

Recently, demographic studies have proposed that the decline of some demographic parameters may co-occur with the increase of others in populations across the geographic range of plant species or over time in a plant population, a phenomenon called ‘demographic compensation’ (Doak and Morris 2010; García-Camacho *et al*. 2012; Sheth and Angert 2018). For instance, natural populations of *Silene* spp. and *Polygonum* spp. in the southern boundary of the tundra had lower survival but higher growth at warmer temperatures than those in northern habitats (Doak and Morris 2010). In this case, the decline of survival could be compensated for by the increase in growth, which contributes to population persistence. Although the relative significance of local adaptation and phenotypic plasticity in the demographic compensation is still controversial (Villellas *et al*. 2015), temporal demographic compensation, mainly shaped by phenotypic plasticity, is suggested to affect population persistence in response to future environmental changes (Doak and Morris 2010; Sheth and Angert 2018).

Notably, plant species often exhibit intraspecific variation in phenotypic plasticity (Sultan 2000; Miner *et al*. 2005; Valladares *et al*. 2014; Matesanz and Ramírez-Valiente 2019). Plant populations in geographically separated mountains are also expected to have differential phenotypic plasticity because of the genetic differences between them induced by limited gene flow (Aegisdóttir *et al*. 2009; Morente-López *et al*. 2018). Demographic parameters, including age-specific survival and reproduction, determine the extinction risk of the natural populations (Caswell 2000). Thus, for conservation purposes, it is of particular interest to examine whether high mountain plant populations have variable plasticity in demographic parameters and whether the plasticity shows a pattern of demographic compensation. The demographic compensation hypothesis predicts that plasticity of different demographic parameters in a population would have opposite directions in response to environmental changes. In addition, like a negative correlation between survival and growth across plant populations (Villellas *et al*. 2015), the plasticity of survival might correlate with the plasticity of growth across natural populations since average trait values may correlate with the magnitudes of trait plasticity (Auld *et al*. 2009).

In this study, we examined the patterns of phenotypic plasticity of the subalpine plant species, *Primula farinosa* subsp*. modesta*, a rosette-forming perennial herbaceous plant found in southern Korea and Japan (Yamazaki 2003). We conducted a growth-chamber experiment to assess plastic responses of *P. farinosa* at the early developmental stage in response to temperature and nitrogen deposition. Plants at the early developmental stage likely have a lower tolerance to environmental stresses so that they might be more vulnerable than adult plants to future environmental changes.

We additionally inspected the demography of *P. farinose* populations in the field to evaluate the results of the growth-chamber experiment. First, as a way to assess whether the plasticity of *P. farinosa* seedling affects population demography, we conducted an elasticity analysis to estimate the contribution of plant life stages to the projected population growth rate quantitatively. The seedling establishment and growth are shown to have a considerable contribution to the population persistence in some alpine plant species (Schlag and Erschbamer 2000; Forbis 2003; Kim and Donohue 2013), but no information is available for *P. farinosa*. Secondly, to confirm that plant performance in the control treatment was comparable to that in the field environment, we examined whether population differentiation observed in the field was reproducible in the control treatment. Specifically, the following questions were addressed: (i) Does the early developmental stage contribute to the population growth rate in *P. farinosa* under natural conditions? (ii) Do the temperature and nitrogen treatments affect the survival and growth? (iii) Do populations exhibit differential phenotypic plasticity to environmental treatments? (iv) Do the plastic responses of survival and growth have an opposite direction and negatively correlate across populations?

## Materials and Methods

### Study species

*Primula farinosa* is a perennial polycarpic herb inhabiting the grassland and mountains in Northern Europe and Northern Asia (Hambler and Dixon 2003). It produces heterostylous flowers with short (thrum-eyed flower) or long style (pin-eyed flowers) which exhibits incompatibility within the same flower morph (Arnold and Richards 1998). Competition with co-occurring plant species is suggested as a major factor decreasing the size of natural populations in Sweden grasslands (Lindborg and Ehrlén 2002). *Primula farinosa* in Korea occurs mainly in open habitats of subalpine areas higher than 1000 m above sea level. Korean populations had relatively low genetic diversity within but high genetic differentiation among natural populations compared to those of other *Primula* species (Chung *et al*. 2013). While climate change is often hypothesized to threaten *P. farinosa* natural populations (Chung *et al*. 2013), its demography and phenotypic plasticity are largely unknown.

### Study sites and demographic survey

**Figure 1.**
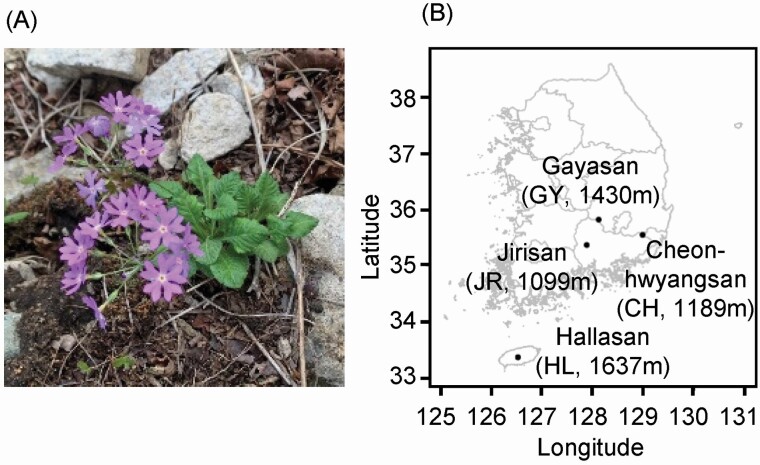
A photograph of *Primula farinosa* (A) and study populations (B) in the southern Korean Peninsula. Names and altitudes of the source populations are given in parenthesis.

We evaluated four natural populations of *P. farinosa* at the following four mountains in the southern part of the Korean Peninsula: Jirisan (JR), Cheonhwangsan (CH), Gayasan (GY) and Hallasan (HL) (Fig. 1). The plants were located in the grasslands near the mountaintops at the CH, GY and HL sites and between rocks of a steep slope at the JR site.

We established three 1 × 1 m quadrats, each with approximately 10–30 individual plants, for each population in June 2016. Regular censuses were conducted at the beginning (April to May) and end (August to September) of the growing season from 2016 to 2018, so a total of six censuses were conducted. We marked all the plants in each quadrat, counted the number of new seedlings, and measured plant traits including leaf number, rosette diameter, census-to-census survivorship and fruit number in each census. A total of 225 individuals in 2016 (CH population: 48; GY population: 63; HL population: 70; JR population: 44) and 213 individuals in 2017 (CH population: 45; GY population: 53; HL population: 79; JR population: 36) were recorded.

To estimate the projected population growth rate (λ), transition matrices were constructed for each population. Following Lindborg and Ehrlén (2002), life stages in plants were defined as seedlings that were less than 1 year old, small vegetative plants (SVs), large vegetative plants (LVs) and reproductive adults (RAs). The vegetative plants were classified into small or large based on the minimum size of a flowering individual for each population. The seed life-cycle stage was not included in the model because of a lack of information, so it is combined with the seedling stage following Caswell (2000). A previous demographic study for *P. farinose* populations showed that combining the seed and seedling stage in the matrix model had a small effect on the population growth rate (Lindborg and Ehrlén 2002). As we could not distinguish seedlings from SVs in 2016, we calculated average transition probability using data in 2017 and 2018

### Plant responses to temperature and nitrogen deposition

Seeds from 15 maternal plants of each natural population were used for this experiment. After soaking in 100 mM gibberellic acid (Sigma, Darmstadt, Germany) solution for 24 h to stimulate germination, seeds were sprinkled into flats filled with commercial soil (ShinSung Mineral Co., Kyeonggi-do, Korea) and decomposed granite (1:1.5 v/v). The flats were placed in a plant growth chamber (Hanbaek Co., Kyeonggi-do, Korea) for 1 month at 20 °C and a 12/12-h light/dark photoperiod with 100 μmol s^−1^ m^−2^ photosynthetically active radiation (PAR) intensity. The seedlings with three or four true leaves were transplanted 4 weeks later into individual plastic pots (90 mm diameter) filled with the same soil as that used for seed germination. Because of a low germination rate, 59 plants from the CH and HL populations, 57 plants from the JR population and 56 plants from the GY population, i.e. 231 plants, were used in the experiment. Transplanted plants were transferred to customized walk-in chambers at 20 °C and a 12/12-h light/dark photoperiod with 200 μmol s^−1^ m^−2^ PAR intensity. The walk-in chamber was equipped with an automated cooling/heating system and air fans to maintain temperature. Environmental treatments were applied to plants 1 week after transfer.

To evaluate the effects of temperature, nitrogen deposition and their interaction, we conducted a full factorial design experiment with the following four treatment combinations: high temperature with nitrogen deposition, high temperature without nitrogen deposition, control temperature with nitrogen deposition and control temperature without nitrogen deposition. One seedling from each of 15 maternal genotypes was randomly assigned to one of four environmental treatments. Number of individuals per treatment is presented in Supporting Information—**Table S1**. We set 17 °C as the control temperature for plant growth because the average temperature in the growing season, from May to September, was 17.34 °C at a meteorological station (1089 m a.s.l.) near the JR site (Korea Meteorological Administration). In addition, *P. farinosa* grows best between 17 and 20 °C during the early developmental stage and is damaged beyond this temperature range (J. Yang, Korea National Arboretum, pers. comm.). The treatments with high temperature were set at 22 °C based on the prediction that the mean temperature in the Korean Peninsula will increase 2.9–4.7 °C above the current temperature (1981–2010) at the end of this century (Suh *et al*. 2016). For treatments with nitrogen, 20 mL of 3 mM ammonium chloride (NH_4_Cl, Sigma) and 20 mL of 0.3 mM potassium nitrate (KNO_3_, Sigma) were added to each pot once a week. A preliminary study showed that the amounts of ammonium and nitrate in the field soil were equivalent to those added to each pot (H. Jeong, unpublished data). To avoid any abrupt increase in nitrogen, we chose to add small amounts of nitrogen compounds repeatedly so that the total amount of nitrogen accumulated towards the end of the experiment. Plants were maintained in two walk-in chambers at 17 or 22 °C for 11 weeks, during which ammonium chloride and potassium nitrate solution were applied. Plants inside the chamber were randomly arranged, and they were rotated between chambers every 4 weeks.

### Trait measurements

In natural conditions, *P. farinosa* seeds germinate from April to May, the beginning of the growing season (H. Jeong, pers. obs.). Yearly survival probability (1 for surviving and 0 for dead plants) of individual seedling was determined from the field observation of whether a seedling survived from the beginning of one growing season to the beginning of the next growing season. Given that all seedlings occurred at the beginning of the growing season, the rosette diameter and leaf number measured at the end of the growing season were used to estimate the seedling growth for 1 year.

After 11 weeks of experimental treatments in walk-in chambers, we recorded survival probability (1 for surviving and 0 for dead plants) from the beginning to the end of experimental treatments, counted the number of leaves and measured the diameter of the rosettes. We also measured the carbon assimilation rate and leaf chlorophyll content since both traits were suggested to correlate with plant growth (Arntz *et al*. 1998; Metcalf *et al*. 2006). The carbon assimilation rate was measured using the LCpro-SD portable photosynthesis system (ADC Bioscientific, Ltd., Hoddesdon, UK). A leaf that was attached to the plant body was placed in the leaf chamber (6.25 cm^2^) with an LED light source. Light intensity was set to 1044 μmol s^−1^ m^−2^ under which *P. farinosa* showed maximum carbon assimilation rate (H. Jeong, unpublished data). Ambient CO_2_ (420–450 ppm) with a flow rate of 100.4 mL min^−1^ was supplied to the leaf chamber. All measurements were made from 9:00 am to 3:00 pm for 3 days. Leaf chlorophyll content was measured using a SPAD-520 Plus chlorophyll meter (Spectrum Technologies, Aurora, IL, USA). The SPAD value is exponentially correlated with chlorophyll content (Uddling *et al*. 2007). Not all plants had leaves with sufficient size for the measurements, so 114 plants were used to measure the carbon assimilation rate, and 157 plants were used to measure the leaf chlorophyll content.

### Statistical analysis

We used the R statistical package ver. 3.2.4 (R Foundation for Statistical Computing, Vienna, Austria) for all statistical analyses. The deterministic population growth rate and its standard error for each population were calculated using the delta method based on the first-order Taylor series (Alvarez-Buylla and Slatkin 1994; Caswell 2000). *T*-tests were conducted to determine whether the deterministic population growth rate was different from one, the criterion for the increase or decrease of the population size, and the statistical significance was evaluated based on Bonferroni adjustment for multiple comparisons. Elasticities of the matrix elements were calculated using popdemo package (Stott *et al*. 2012). We examined whether the seedling survival and size measured at the end of the growing season differed in natural populations and whether such a pattern was reproduced in plants grown in the growth-chamber environment. Field data from 2017 and 2018 were combined to increase the sample size. To compare the yearly survival of seedlings among *P. farinosa* natural populations, binary survival for 1 year was analyzed using a logistic model with a binomial distribution. The model included population as an independent variable and its significance was evaluated using χ ^2^ values from the likelihood-ratio test. One-way analyses of variance (ANOVA) with the population as fixed factor followed by the *post-hoc* Tukey’s multiple comparison tests were conducted to compare leaf number and rosette diameter of seedlings among populations in natural environments. The same models were used to examine population differentiation in the leaf number and rosette diameter for plants grown in the control temperature/ no nitrogen treatment.

Effects of environmental treatments on the measured traits were assessed using similar models for the analyses of natural populations. The binary survival from the beginning to the end of experimental treatments was evaluated using a logistic model with a binomial distribution, and the responses of morphological and physiological traits were analyzed using ANOVA. The models included the source population, temperature, nitrogen and their two-way interactions as fixed factors. The three-way interaction was removed from the model following Allison (1999) because the inclusion of three-way interaction caused some independent variables to perfectly separate the survival response. ANOVA for the other measured traits did not include the three-way interaction because it was not statistically significant when included in the model. The leaf number, SPAD value and carbon assimilation rate were log-transformed to meet normality assumptions. The analysis of the binary survival showed a significant population by nitrogen interaction (see Results). To interpret such interaction, a two-by-two contingency table with the binary survival and nitrogen treatment was constructed for each population. The independence of variables was examined using Fisher’s exact test, and *P* values were adjusted based on Bonferroni correction for multiple comparisons. To interpret temperature by population or temperature by nitrogen interactions for morphological and physiological traits, differences in the least squared means between temperature treatments were evaluated for each population or each nitrogen treatment with Bonferroni correction for multiple comparisons.

To evaluate phenotypic plasticity in environmental treatments, we used a modified relative distance plasticity index (RDPI) for each trait following Valladares *et al*. (2006). The relative distance between trait values was calculated as the difference of trait value divided by the sum of trait values between all pairs of individuals that were grown in different environments. For the relative distance, the difference of trait values was used instead of the absolute values of the difference to consider the direction of plastic responses. The RDPI for high temperature was the average of the relative distances between temperature treatments with and without nitrogen deposition, and the RDPI for nitrogen deposition was the average of the relative distances between nitrogen treatments in low and high temperatures. One-way ANOVA and *post-hoc* Tukey’s mean comparison tests were conducted to compare RDPI among natural populations. Regression analysis was conducted to explore the relationship between the plasticity index of survival and the RDPI of measured traits. The plasticity of survival was calculated as the difference of average survival between treatments divided by the sum of average survival for each population. Treatment was included in the model to control the differences between temperature and nitrogen treatments.

## Results

### Plant performances in natural habitats

**Figure 2.**
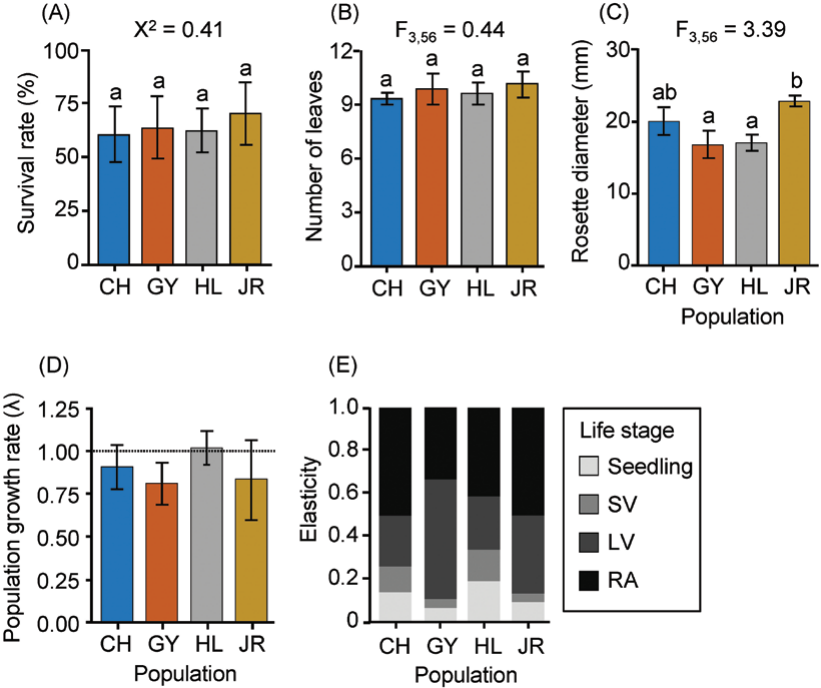
Results of field survey in four *Primula farinosa* natural populations. Averages and standard errors of (A) yearly survival, (B) leaf number and (C) rosette diameter are given. The leaf number and rosette diameter were measured at the end of the growing season. χ ^2^ value for survival and *F* ratios (population) for the other traits are given. Letters indicate statistically significant differences among populations at the 0.05 level based on Tukey’s adjustment for multiple comparisons. Population growth rate at a 95 % confidence interval (D) and elasticities (E) were calculated based on the combined transition matrix during 2 years (see **Supporting Information—Table S2**).

The seedlings of *P. farinosa* natural populations exhibited similar yearly survival rates with 74–81 % in the natural conditions (χ ^2^ = 0.41, *P* = 0.938) (Fig. 2A). In contrast, the rosette diameter measured at the end of growing season differed among natural populations (*F*_3,56_ = 3.39, *P* < 0.05) (Fig. 2C). In particular, seedlings of JR population had larger rosettes than those of the HL and GY population.

The projected population growth rate (λ) differed among natural populations. The λ value was not different from one with statistical significance in the CH (*z* = 1.55, adjusted *P* = 0.24) and HL (*z* = −0.24, adjusted *P* = 1) population, indicating those *P. farinosa* populations likely maintain their current population size. The λ value in the GY population was 0.804, which was lower than 1 with statistical significance (*z* = 3.16, adjusted *P* < 0.01) (Fig. 2D). The JR population had λ value (0.826) similar to the GY population, but it was not different from 1 with statistical significance (*z* = 1.47, adjusted *P* = 0.28), probably due to large standard error. The elasticities of large vegetative and reproductive plants were higher than those of seedlings and small vegetative adults. However, the survival and growth of seedlings also contributed 5.2–19.0 % to the λ (Fig. 2E). The contribution of the seedling stage to the λ varied among populations such that the GY and JR populations showed lower elasticity of seedling stage than the other populations.

### Plant performance under environmental treatments

Under control temperature (17 °C) and without nitrogen deposition, *P. farinosa* populations exhibited similar survival rates (χ ^2^ = 4.70, *P* = 0.195). In contrast, rosette diameter differed among populations (*F*_1,48_ = 2.80, *P* < 0.05) such that plants from the JR population grew more vigorously than those from other populations. These results were consistent with the field observations (Fig. 2), whereas they were inconsistent in that plants in the growth chamber exhibited higher survival rates and lower leaf growth than those in the field. Populations produced similar number of leaves in both, the field (*F*_3,56_ = 0.25, *P* = 0.86) and the control temperature and low nitrogen treatment (*F*_3,48_ = 0.80, *P* = 0.50).

**Figure 3.**
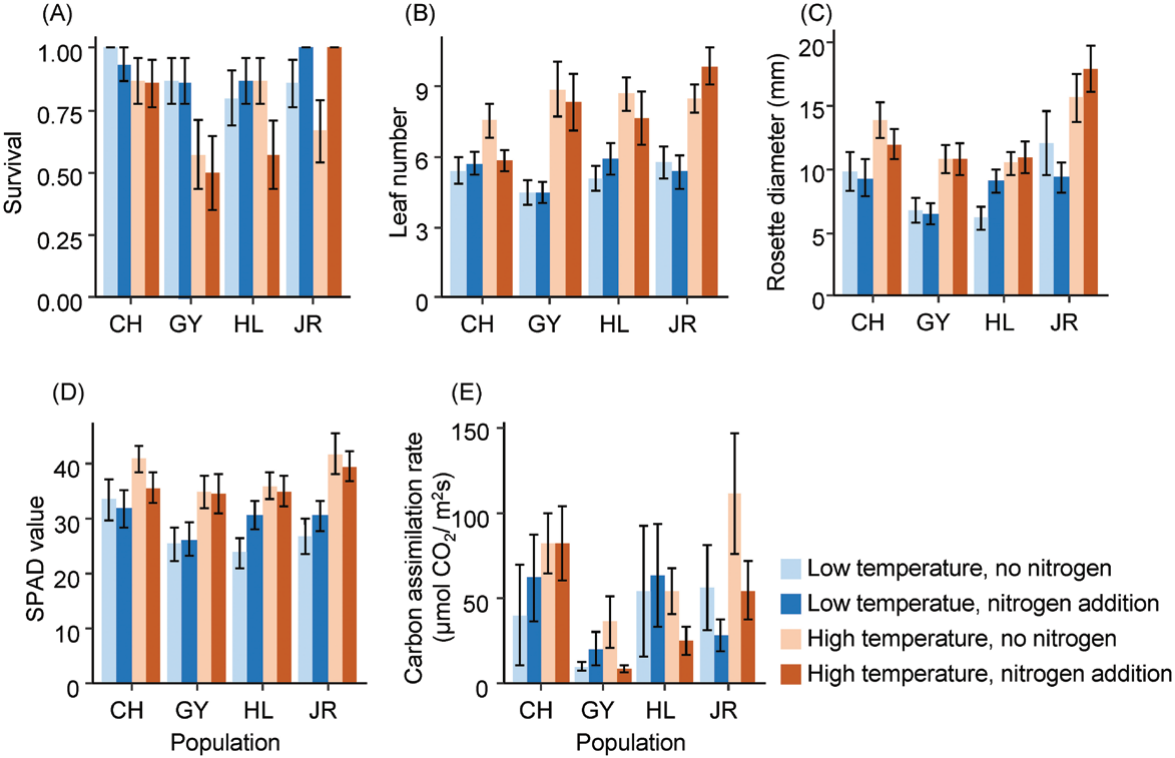
Effects of source population, growth temperature and nitrogen deposition on (A) survival, (B) leaf number, (C) rosette diameter, (D) SPAD value and (E) carbon assimilation rate. See Table 1 for the results of the significance test and Fig. 1 for the names of the natural populations tested.

The high temperature treatment reduced seedling survival similarly for all populations (Table 1, Fig. 3A). Nitrogen treatment effect differed among the four natural populations as indicated by a significant population by nitrogen interaction (Table 1). Fisher’s exact test showed that the JR population exhibited higher survival under high nitrogen condition (adjusted *P* < 0.05), while the other populations did not (CH population, adjusted *P* = 1.00; GY population, adjusted *P* = 1.00; HL population, adjusted *P* = 1.00).

**Table 1.** Results of ANOVA to compare survival and plant traits among *Primula farinosa* populations and treatments. Chi-square values are given for survival and *F* values are given for the other traits. All traits except survival and rosette diameter were log-transformed to meet normality assumptions. Sample sizes are presented in parenthesis. Pop, source population; Temp, temperature treatment; Nit, nitrogen treatment. ^†^*P* < 0.1, *P < 0.05, **P < 0.01, ***P < 0.001.

	Pop	Temp	Nit	Pop × Temp	Pop × Nit	Temp × Nit
Survival (231)	14.18**	10.17**	2.65	1.55	12.27**	0.71
Rosette diameter (191)	9.61***	33.97***	0.00	1.02	0.78	0.05
Leaf number (191)	1.78	61.11***	0.20	3.06*	0.08	0.99
SPAD (157)	2.34^†^	35.52***	0.25	0.66	0.10	2.95^†^
Carbon assimilation rate (112)	5.48**	3.91^†^	1.01	0.68	0.42	3.52^†^

While the high temperature treatment reduced seedling survival, it stimulated seedling growth. Plants produced more leaves with longer length and higher chlorophyll contents at high temperatures than at control temperature (Table 1, Fig. 3). Notably, the effect of temperature on leaf production differed among populations. Plants from the GY (*t* ratio = 4.89, adjusted *P* < 0.001), HL (*t* ratio = 3.45, adjusted *P* < 0.01) and JR (*t* ratio = 5.25, adjusted *P* < 0.001) populations produced more leaves when grown in the high temperature, but those from the CH (*t* ratio = 1.86, adjusted *P* = 0.26) population did not.

The effect of temperature on the carbon assimilation rate depended on nitrogen treatment as indicated by a marginally significant temperature and nitrogen interaction (Table 1). Temperature treatment increased the carbon assimilation rate only when the plants were grown in high nitrogen soil (*t* ratio = 2.67, adjusted *P* < 0.05), and the effect of temperature was not detected in plants in low nitrogen soil (*t* ratio = 0.08, adjusted *P* = 1.00).

### Phenotypic plasticity in response to the environmental treatments

**Figure 4.**
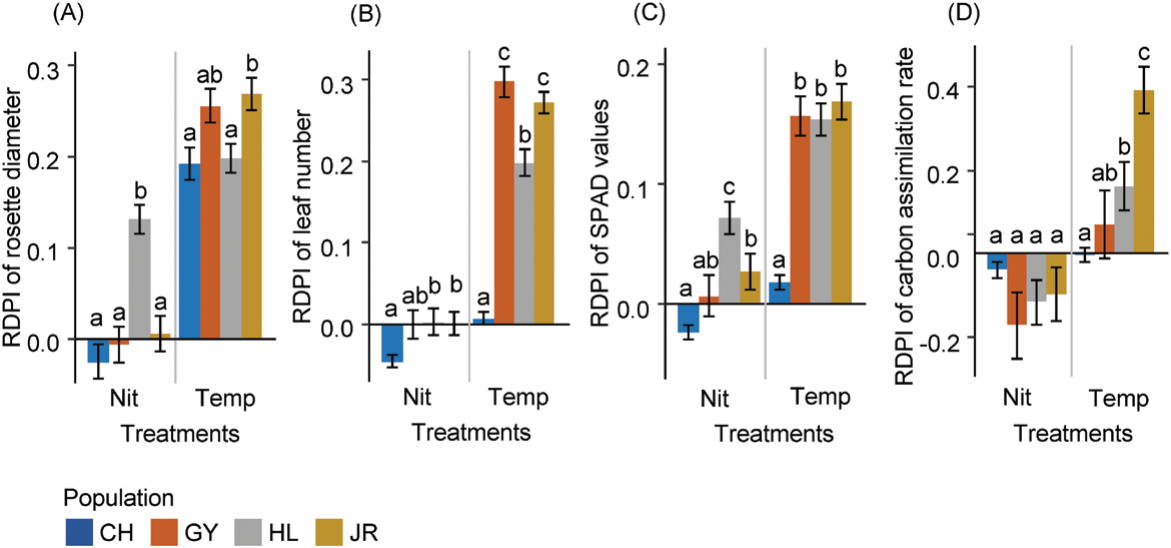
RDPI of plant traits of four *Primula farinosa* natural populations. Average RDPI values and standard errors of (A) rosette diameter (B) leaf number, (C) SPAD value, and (D) carbon assimilation rate are given. Letters indicate statistically significant differences among species at the 0.05 level based on Tukey’s adjustment. Temp, temperature treatment; Nit, nitrogen treatment.

**Figure 5.**
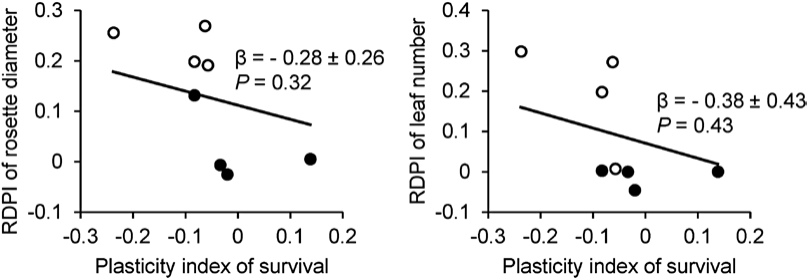
Results of regression analysis to examine relationship between the plasticity index of survival and average RDPI of plant traits. Regression coefficient and its standard error are given. Filled circles represent RDPI in response to the high temperature and open circles represent RDPI in response to the nitrogen deposition. Solid line is a regression line across environmental treatments.

In response to the environmental treatments, *P. farinosa* populations exhibited differential RDPIs in all measured traits except in the carbon assimilation rate responding to the nitrogen treatment (Fig. 4). The CH population exhibited the lowest RDPI of the leaf production, rosette diameter, SPAD value and carbon assimilation rate compared to the other populations. Although the nitrogen treatment did not affect average trait values (Table 1), natural populations exhibited differential RDPIs in response to the nitrogen treatment (Fig. 4). In particular, the CH population exhibited negative RDPIs of traits, indicating that plants in the high nitrogen soil exhibited lower trait values than those in the low nitrogen soil. No relationship was detected between the plasticity index of survival and the average RDPI of the measured traits (Fig. 5).

## Discussion

In response to high temperature, the survival of *P. farinosa* seedlings decreased but the leaf number, rosette diameter and leaf chlorophyll content increased. Nitrogen deposition increased the survival of one natural population, but did not affect other populations. Notably, populations exhibited differential plasticity to environmental treatments. As the seedling stage had a moderate amount of elasticity, i.e. the contribution to the population growth rate, our results suggest that phenotypic plasticity would contribute to the persistence of *P. farinosa* populations in the future, but the degree of contribution would differ among populations.

### Plastic response of seedling survival

Increasing temperature affected the survival of *P. farinosa* seedlings such that high temperature decreased seedling survival. In contrast, nitrogen deposition increased seedling survival in the JR population and did not affect the survival of the other populations. Considering the contribution of the seedling stage to the population growth rate (Fig. 2), differential seedling survival of *P. farinosa* natural populations would affect population persistence in the future. For instance, field survey showed that in the GY and JR populations, the population growth rate was estimated to be <1, indicating that their population size is currently decreasing. Although high temperature decreased seedling survival in both populations, nitrogen deposition increased seedling survival of the JR population but had no effects on the seedling survival of the GY population. Thus, environmental changes would potentially accelerate the extinction risk of the GY population, but ameliorate the decline of the JR population.

Nitrogen deposition has been reported to alter diverse soil characteristics like pH and availability of nutrients (Porter *et al*. 2013). In this study, the early survival of the JR population of *P. farinosa* increased in response to nitrogen deposition. This is in contrast to a previous hypothesis that the growth and survival of alpine and subalpine plants are insensitive to soil nitrogen contents (Bowman *et al*. 2006). Based on this hypothesis, most previous studies have focused on the plausible invasion of the so called fast growing plant species into alpine or subalpine areas and their consequences on competition among plant species and on species richness (Bowman *et al*. 2006; He *et al*. 2011). Our results suggest that nitrogen deposition could influence the important demographic parameter of survival during the early developmental stage, and such an effect would be species- and population-specific.

### Plastic responses of plant traits

Although high temperature decreased the early survival of *P. farinosa*, it increased leaf production, chlorophyll content and carbon assimilation rate (Table 1, Fig. 3). Similar responses have also been reported in other alpine plant species (Arft *et al*. 1999; Byars *et al*. 2007; Kudernatsch *et al*. 2008; Kim and Donohue 2013), suggesting that the opposite plastic responses of survival and growth to increasing temperature might be common. As growth and survival are important demographic parameters that affect population growth rate, increased growth of plants like *P. farinosa* at high altitudes could ameliorate the decline of population size due to high mortality under increasing temperature (Villellas *et al*. 2015).

Notably, phenotypic plasticity indexes of leaf number and rosette diameter differed among natural populations in response to environmental treatments. Thus, although stimulated growth by increasing temperature would contribute to population persistence, the degree of contribution would vary among natural populations of *P. farinose*. The role of plasticity in growth for population persistence is likely population-specific. In this study, two analytical methods were used to detect population differentiation in phenotypic plasticity, and the results were different. ANOVA results showed that only leaf production had a statistically significant population by temperature interaction (Table 1), an indication of differential population responses to temperature treatment. In contrast, RDPI analysis revealed that all measured traits exhibited variable phenotypic plasticity among natural populations (Fig. 4). Although ANOVA has been widely used to detect population differentiation, recent studies suggested that a phenotypic plasticity index like RDPI is more appropriate for a comparative study since it quantitatively estimates phenotypic plasticity (Valladares *et al*. 2006).

Given the differential plasticity among natural populations, detecting patterns of variation was suggested to be crucial for a more precise prediction of the species range under changing environments (Valladares *et al*. 2014; Matesanz and Ramírez-Valiente 2019). Demographic studies showed that survival and growth had a negative correlation across populations in the geographic range of plant species (Doak and Morris 2010; Villellas *et al*. 2015); therefore, populations with high mortality exhibit more vigorous growth. Accordingly, the plasticity of survival was expected to correlate with the plasticity of growth-related traits across populations. However, no such correlation was detected (Fig. 4), indicating that plastic responses of survival and growth-related traits of populations would be independent. Alternatively, this result might be due to the limited number of populations we examined (Makin and de Xivry 2019). A future study including more populations would provide more valid conclusion.

The effect of high temperature on the carbon assimilation rate was observed only when plants were grown at high nitrogen treatment. Similar to their interactive effects on biodiversity (Porter *et al*. 2013), the effect of nitrogen deposition on plant traits may depend on temperature conditions. Since no such interaction was detected in the leaf production and leaf growth (Table 1), it is still inconclusive whether temperature and nitrogen deposition interactively affect plant growth.

While opposite plastic responses to temperature in seedling survival and growth were observed in this study, it is inconclusive whether such plasticity has the power to rescue threatened alpine plant species (Sheth and Angert 2018; Campbell 2019). We examined the plasticity of plants at the early developmental stage, but its contribution to the population persistence might be limited considering the relatively high elasticity of the adult stage. In addition, we did not have information on the major demographic parameter of fecundity, although fecundity may exhibit a pattern of demographic compensation with other demographic factors (Villellas *et al*. 2015). Lastly, changing environments would also affect competition among plant species and pollinators, consequently influencing growth and fecundity (Ehrlén *et al*. 2005; Inouye 2020). We are currently conducting a long-term field demographic study that will provide more information on the temporal plastic responses of demographic parameters and their effects on population persistence.

## Supporting Information

The following additional information is available in the online version of this article—

**Table S1.** The number of *P. farinosa* individuals assigned to each experimental treatment.

**Table S2.** The transition matrices and elasticity matrices of *Primula farinosa* populations from 2016 to 2018. The transition probabilities and elasticities of four life stages are presented. SV, small vegetative plant; LV, large vegetative plant; RA, reproductive adult. The transition of RA to seedling was calculated as the number of new seedlings per plant.

plab061_suppl_Supplementary_Material

## Data Availability

The dataset is available in Figshare: https://doi.org/10.6084/m9.figshare.16573079.v1.

## Notes

**Natural History and Conservation. Chief Editor:** F. Xavier Picó
