# Supplementary material for: Differential plastic responses to temperature and nitrogen deposition in the subalpine plant species, Primula farinosa subsp. modesta
Source: AoB Plants. 2021 Sep 19;13(5):plab061. doi: 10.1093/aobpla/plab061 (PMC8501906; doi:10.1093/aobpla/plab061)
Supplement: plab061_suppl_Supplementary_Material [file plab061_suppl_supplementary_material.pdf]

Supporting information

Table S1. The number of *P. farinosa* individuals assigned to each experimental treatment.

Population	Experimental condition			
	Control temperature, no nitrogen addition	High temperature, no nitrogen addition	Control temperature, nitrogen addition	High temperature, nitrogen addition
CH	15	15	15	14
GY	15	14	15	12
HL	15	15	15	14
JR	14	15	14	14
Total	59	59	59	54

Table S2. The transition matrices and elasticity matrices of *Primula farinosa* populations from 2016 to 2018. The transition probabilities and elasticities of four life stage are presented. SV, small vegetative plant; LV, large vegetative plant; RA, reproductive adult. The transition of RA to seedling was calculated as the number of new seedlings per plant.

	Population transition matrix				Elasticity matrix			
Cheonhwangsan population (CH)								
from / to	Seedling	SV	LV	RA	Seedling	SV	LV	RA
Seedling	0.000	0.000	0.000	0.238	0.000	0.000	0.000	0.061
SV	0.550	0.277	0.141	0.089	0.042	0.042	0.027	0.025
LV	0.184	0.379	0.399	0.089	0.019	0.080	0.107	0.035
RA	0.000	0.044	0.252	0.705	0.000	0.015	0.107	0.441
Gayasan population (GY)								
Seedling	0.000	0.000	0.000	0.141	0.000	0.000	0.000	0.026
SV	0.292	0.092	0.025	0.000	0.010	0.003	0.012	0.000
LV	0.292	0.458	0.581	0.394	0.016	0.022	0.418	0.123
RA	0.000	0.000	0.140	0.481	0.000	0.000	0.149	0.222
Hallasan population (HL)								
Seedling	0.000	0.000	0.000	0.296	0.000	0.000	0.000	0.095
SV	0.170	0.243	0.137	0.146	0.018	0.033	0.034	0.052
LV	0.456	0.316	0.367	0.220	0.037	0.043	0.090	0.079
RA	0.267	0.316	0.356	0.571	0.040	0.061	0.125	0.293
Jirisan population (JR)								
Seedling	0.000	0.000	0.000	0.201	0.000	0.000	0.000	0.049
SV	0.000	0.296	0.037	0.000	0.000	0.008	0.015	0.000
LV	0.292	0.472	0.489	0.125	0.049	0.015	0.218	0.086
RA	0.000	0.000	0.309	0.626	0.000	0.000	0.135	0.425
